# Patient-specific metal implants for focal chondral and osteochondral lesions in the knee; excellent clinical results at 2 years

**DOI:** 10.1007/s00167-020-06289-7

**Published:** 2020-10-06

**Authors:** Johannes Holz, Tim Spalding, Tarek Boutefnouchet, Pieter Emans, Karl Eriksson, Mats Brittberg, Lars Konradsen, Clemens Kösters, Peter Verdonk, Magnus Högström, Martin Lind

**Affiliations:** 1 OrthoCentrum Hamburg, Hansastrasse 1-3, 20149 Hamburg, Germany; 2 Department Trauma and Orthopaedics, University Hospital Coventry, Clifford Bridge Road, Coventry, UK; 3 Department of Orthopaedics, Maastricht UMC+, P. Debyelaan 25, 6229 HX Maastricht, The Netherlands; 4 Department of Orthopaedics, Stockholm South Hospital, Karolinska Institutet, Sjukhusbacken 10, 118 83 Stockholm, Sweden; 5 Cartilage Research Unit, University of Gothenburg, Region Halland Orthopaedics, Kungsbacka Hospital, 434 80 Kungsbacka, Sweden; 6 Department of Orthopaedic Surgery, Bispebjerg Hospital, Bispebjerg Bakke 23, 2400 Bispebjerg, Denmark; 7 Clinic for Orthopaedics, Trauma and Hand Surgery, Maria-Josef-Hospital Greven, Lindenstr. 29, 48268 Greven, Germany; 8 Orthoca, AZ Monica Hospitals, Harmoniestraat 68, 2018 Antwerp, Belgium; 9 Sports Medicine Umeå AB and Orthopedics, Department of Surgical and Perioperative Sciences, Umeå University, 901 87 Umeå, Sweden; 10 Deptartment of Orthopedics, Aarhus University Hospital, Palle Juul Jensens Boulevard 99, 8200 Århus, Denmark

**Keywords:** Focal cartilage lesion, Focal knee resurfacing, Metal implants, Clinical outcomes, Customised, Personalized

## Abstract

**Purpose:**

Surgical treatment options for the management of focal chondral and osteochondral lesions in the knee include biological solutions and focal metal implants. A treatment gap exists for patients with lesions not suitable for arthroplasty or biologic repair or who have failed prior cartilage repair surgery. This study reports on the early clinical and functional outcomes in patients undergoing treatment with an individualised mini-metal implant for an isolated focal chondral defect in the knee.

**Methods:**

Open-label, multicentre, non-randomised, non-comparative retrospective observational analysis of prospectively collected clinical data in a consecutive series of 80 patients undergoing knee reconstruction with the Episealer® implant. Knee injury and Osteoarthritis Outcome Score (KOOS) and VAS scores, were recorded preoperatively and at 3 months, 1 year, and 2 years postoperatively.

**Results:**

Seventy-five patients were evaluated at a minimum 24 months following implantation. Two patients had undergone revision (2.5%), 1 declined participation, and 2 had not completed the full data requirements, leaving 75 of the 80 with complete data for analysis. All 5 KOOS domain mean scores were significantly improved at 1 and 2 years (*p* < 0.001–0.002). Mean preoperative aggregated KOOS4 of 35 (95% CI 33.5–37.5) improved to 57 (95% CI 54.5–60.2) and 59 (95% CI 55.7–61.6) at 12 and 24 months respectively (*p* < 0.05). Mean VAS score improved from 63 (95% CI 56.0–68.1) preoperatively to 32 (95% CI 24.4–38.3) at 24 months. The improvement exceeded the minimal clinically important difference (MCID) and this improvement was maintained over time. Location of defect and history of previous cartilage repair did not significantly affect the outcome (*p* > 0.05).

**Conclusion:**

The study suggests that at 2 years, Episealer® implants are safe with a low failure rate of 2.5% and result in clinically significant improvement. Individualised mini-metal implants with appropriate accurate guides for implantation appear to have a place in the management of focal femoral chondral and osteochondral defects in the knee.

**Level of evidence:**

IV.

## Introduction

Focal chondral and osteochondral lesions are a significant cause of morbidity and can have a clinical impact similar to end-stage osteoarthritis, with an important socio–economic burden [15, 17]. Lesions are likely to progress to bifocal disease and pan-articular osteoarthritis [4, 7, 29].

Available methods for the management of focal chondral and osteochondral defects in the knee include cell-based regenerative procedures, bone marrow stimulation techniques including microfracture or scaffold augmented microfracture, and osteochondral grafting procedures which can be autograft or allograft [3]. Osteochondral allograft reconstruction (OCA) is highly cost-effective [30, 31] but the procedure is limited by the scarcity of fresh grafts and in some countries use is restricted by law [11, 46]. Patient age for all such options is a significant restrictive factor since transplanted cells and marrow cells tend to lose regenerative potential with increasing age [9, 21]. The outcome of unicompartmental knee arthroplasty for focal chondral and osteochondral lesions remains unpredictable, suggesting that this option should be reserved for bone-on-bone disease [14, 22, 36]. Equally, total knee arthroplasty (TKA) for a focal lesion in younger active patients is not considered an acceptable option [25, 28].

Mini-metal and other focal resurfacing techniques have been developed to tackle the so-called “treatment-gap” of patients considered too young and active for arthroplasty and either too old for biological procedures, or who have failed previous articular cartilage repair procedures. Concerns about revision rates with such implants remain present, especially early on, [12, 23] and a recent systematic review reported a conversion rate to TKA of 22% at an average interval of 46 months [10].

An individualised implant, adapted to the patients knee using planning based on Magnetic Resonance Imaging (MRI) and implanted with the benefit of defect specific guides and instrumentation has been developed, intended to improve outcome due to optimised fit, defect cover and congruence.

The aim of the study was to report on the early clinical and functional outcomes in a consecutive series of patients following implantation of an individualised mini-metal implant for isolated focal chondral and osteochondral defects. The clinical relevance of the study is that successful improvement in outcome would expand treatment options for selected patients where currently there are only limited conservative options available. The primary hypothesis was that treatment would result in an increase in KOOS scores at 24 months compared to baseline. A secondary aim was to examine differences in outcome at other recorded time points and explore the effects of previous cartilage repair and the site of implantation.

## Materials and methods

From 2013 to 2017, 80 consecutive patients underwent focal resurfacing with the Episealer Mini-metal implant (Episurf, Sweden) as part of a non-comparative, open-label multicentre study. This study is a retrospective analysis of prospectively collected data.

The study institutions consisted of 9 sites in Europe and involved 11 participating surgeons. Treatment was indicated for patients with symptomatic chondral and osteochondral defects in the knee who had failed conservative treatment and who were suitable for the procedure as determined on specific MRI imaging and satisfactory mapping according to an individualised damage marking report. Contraindications included patients with inflammatory arthritis, age below 35 or above 70, malalignment > 5 degrees, joint space narrowing on weight-bearing x-rays and greater than 50% loss of meniscal tissue. Full inclusion and exclusion criteria are detailed in Table 1.

**Table 1:** Episealer implants patients selection criteria

Inclusion criteria	Exclusion criteria
Age ≥ 18 Focal femoral knee chondral or osteochondral lesion Focal chondral and osteochondral lesions: ICRS III–IVb Symptoms of pain and disability Failed non-operative treatment No radiographic loss of joint space on PA 30**°** Meniscus volume: 50% or more, without extrusion Bone: no deformities, erosions, or deep cystic formations Failed cartilage repair procedure Normal or ICRS I-II opposite articular surface Neutral alignment: defined as < 3 degrees deviation of tibiofemoral mechanical axes Patient expectations appropriate, and no high impact activities	Bone on bone disease Multifocal chondral defects Severe chondral lesion (ICRS III–IV) on opposing surface Previous mini implant focal resurfacing Systemic and/or inflammatory joint disease Joint instability or malalignment that is not correctable at the time of treatment On-going infection in the knee joint Inflammatory arthritis or radiographic osteoarthritis Sensitivity to materials typically used in orthopaedic implants (self-reported or prior exposure) Inadequate bone stock at site of insertion Existing prosthesis in compartment of treatment or opposing surface

Data were collected preoperatively and at 3, 12 and 24 months postoperatively. Baseline demographic and clinical information were recorded, including age, gender, BMI, the American Society of Anaesthesiologists (ASA) score, smoking status, comorbidities, lesion size and location, concomitant knee problems, and prior surgical knee procedures. Clinical outcome forms were completed by the patient on paper or electronically as determined in each centre, and operative data were recorded by the surgeon. Implant failure was defined as development of infection, implant removal and revision. Clinical outcome measures consisted of the Knee injury and Osteoarthritis Outcome Scores (KOOS) and the Visual Analogue Score for pain. Mean overall KOOS4 and individual domain KOOS scores were evaluated against the published minimal clinically important difference (MCID) [40]. Between-group analysis was performed for patients with and without a history of previous cartilage repair, and for location of defect.

**Fig. 1:**
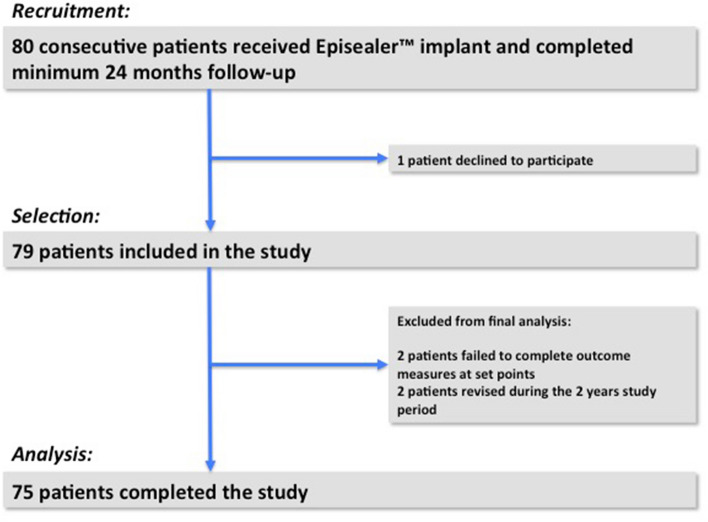
Flow diagram detailing patients’ enrolment and follow-up

One patient declined participation in the study and was, therefore, excluded. Two patients were unable to complete outcome measures at required set time points and 2 patients underwent revision surgery prior to 24 months. An a-priori power analysis was not required since the study aimed to include the entire patient population. Details of the follow-up and numbers in the analysis are outlined in the Flow-Chart in Fig. 1. Complete outcome data for analysis was, therefore, available in 75 patients. The mean age was 48 years (range 27–69) and 41% were male. In 48 patients (64%), Episealer® implants were performed following failed prior articular cartilage repair procedures. Patients’ characteristics, lesion location and previous cartilage treatment are outlined in Table 2. Forty patients were treated with the Episealer condyle Solo implant, 25 with the Episealer condyle Twin implant and 10 were treated for defects on the trochlea (either Episealer Femoral Twin or Episealer Trochlea Solo implants).

**Table 2:** Demographic profile and clinical characteristics

Mean age (range) years	48 (27–69), SD 8.34		
Gender	Female 44, male 31		
BMI (kg/m^2^)	28 (19–41), SD 4.83		
Location of lesion (*n*)	MFC	LFC	Trochlea
	60	5	10
Size of lesion (*n*)	< 3 cm^2^	3–4 cm^2^	> 4 cm^2^
	24	35	16
Prior cartilage lesion treatment (*n*)	48		
Microfracture	31		
Mosaicoplasty	5		
Autologous chondrocyte implantation	4		
Biphasic bioresorbable scaffold (TruFitÔ™ Plug)	3		
Debridement	5		

Previous treatments: Each patient only reported once as per original index procedure, e.g. if microfracture followed by mosaicoplasty then only reported as microfracture

Institutional review board approval was obtained in each participating centre and patients took part in an informed consent process consistent with Good Clinical Practice guidelines. The project was registered in UK (University Hospital Coventry and Warwickshire NHS Trust R&D department Ref: TS448919) and The Netherlands (Maastricht UMC Ref METC 2019-1410) as post-market surveillance approved by the respective universities and formal ethics was not required. IRB and Ethics numbers for remaining centres are Ethik Kommission der Ärztekammer Westfalen-Lippe und der Westfälischen Wilhelms-Universität, Ref: 2020-288-b-S; Universiteit Antwerpen Etisch Comite, Ref: B300201526651; The Scientific Ethical Committee of the Capital Region of Denmark, Ref: H-4-2014-068; Kommission der Ärztekammer Hamburg, Ref: PV7118; Etikprövningsmyndigheten, Ref: Dnr 2019-06268; and the Regionala etikprövningsnämnden i Stockholm, Ref: 2017/1759-31/1.

### The device and surgical procedure

**Fig. 2:**
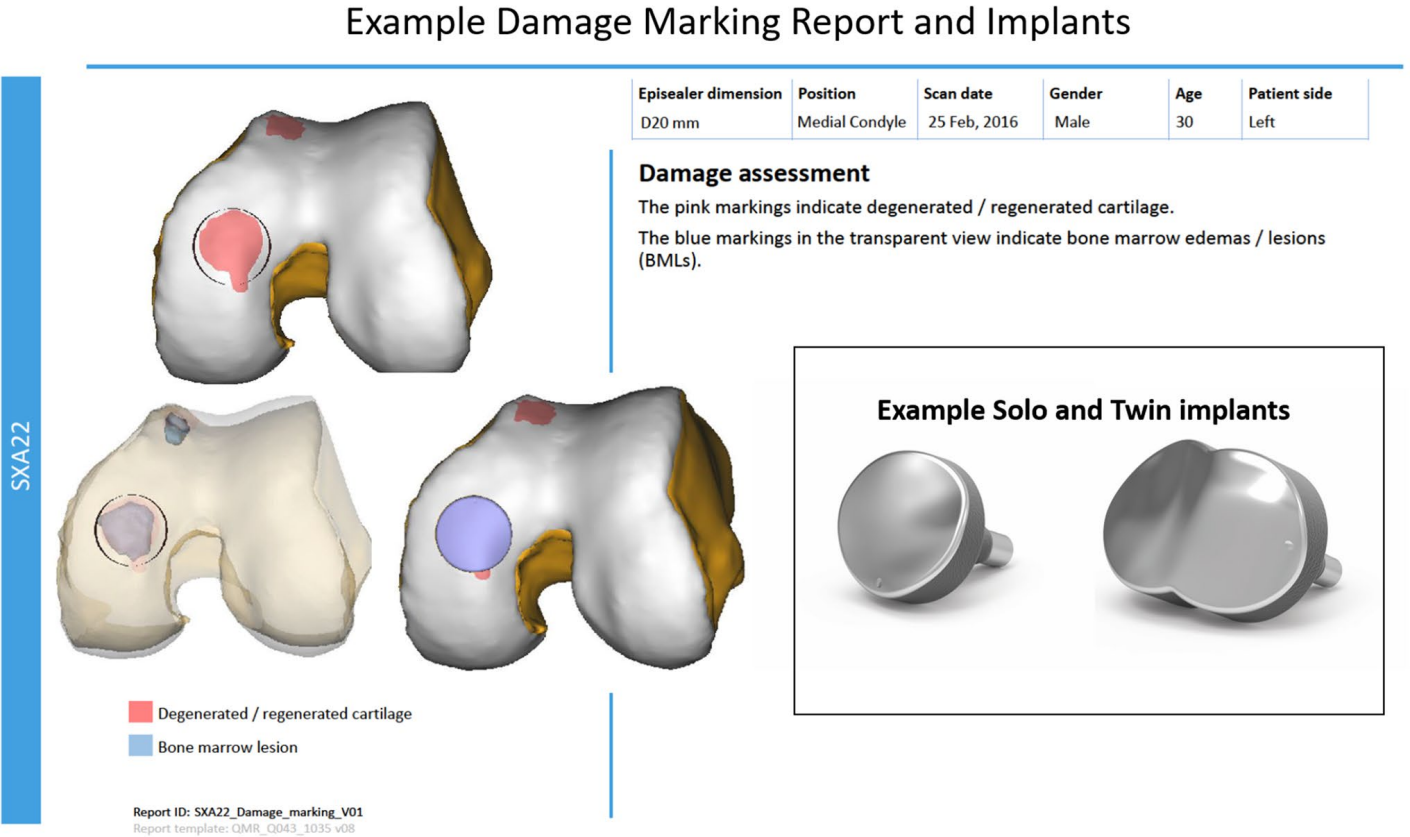
Example of an Episealer™ damage marking report with photographs of sample condylar solo and condylar twin implants

The Episealer® implant (Episurf Medical, Stockholm, Sweden) is manufactured from cobalt chrome with a highly polished articular surface that is individualised to replicate the articular curvature using an interpolating algorithm. The undersurface and sides of the prosthesis are coated with titanium and hydroxyapatite (Eurocoating**,** Part of United Coating Group Anteco SRL Salerno Italy) to achieve optimal biological bone to implant fixation. The Episealer® Solo and Episealer® Trochlea Solo implants are circular with diameters of 12, 15, 17, 20, 25 or 29 mm. The Episealer® Femoral Twin implant consists of two overlapping circles merged into a figure-of-eight configuration with 15, 17, 20 or 25 mm diameters. One or two pins, respectively, on the base help provide immediate fixation (Fig. 2). Both types can be specifically manufactured for the lateral and medial condyles or the trochlea. The planned implant thickness is 4 mm, but thicker components can be produced for deeper lesions.

The individualised design is based on a detailed MRI scan including four 2-dimensional (2D) diagnostic sequences and one 3-dimensional (3D) sequence to allow for a 3D computer reconstruction of the knee articular surfaces. The sequences are specified according to the proprietary software designed by Episurf with each centre undergoing testing and approval of image quality. Patient data are removed with images allocated a patient-specific code known to the referral centre, prior to uploading to an in-house web-based platform. 3D reconstructed images were produced initially by manual segmentation but later by an automated system that identifies and outlines chondral and osteochondral defects. An appropriate Episealer implant is then superimposed on the defect and the resultant Damage Marking Report (DMR) (Fig. 2), together with relevant MR-images, is returned to the surgeon within 4–5 days via the web-platform for approval. Guide instrumentation, specific to the proposed implant, are also designed, and on approval manufacturing of implant and guides is started with delivery to the hospital within approximately 4 weeks.

**Fig. 3:**
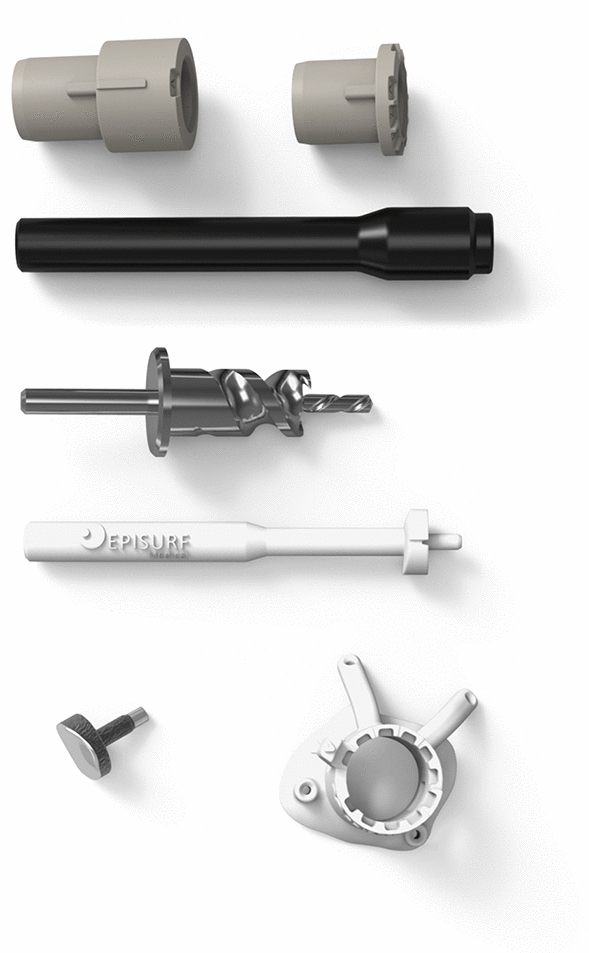
Instrumentation provided for the procedure (from the top): drilling socket and adjustment socket, Epimandrel impactor, Epidrill, Epidummy trial implant, and Epiguide

**Fig. 4:**
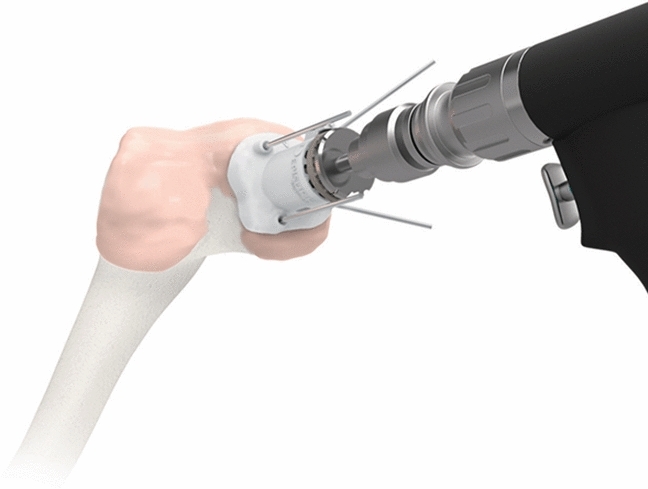
Illustration of operative technique with Epiguide attached to femur with pins providing stable and aligned socket for accurate drilling and preparation for the implant

The set of surgical instruments consist of 6 pieces (Fig. 3). Two of these are individualised—the Epiguide and the Epidummy. Both are 3D printed using polyamide (PA2200). The Epiguide, matching the implant diameter, enables drilling at exactly the correct angle and depth. The expanded lower end matches the healthy cartilage surrounding the defect and when held in place with k-wires it forms a very stable guide (Fig. 4). Variable depth teeth on the upper end of the guide accommodate an inner drill sleeve as an “adjustment socket” such that the drill depth can be incrementally increased in steps of 0.2 mm. An Epidummy, which is an exact replica of the implant on a handle is used to check the final position and drilling depth is advanced using the incremental socket system until the Epidummy sits at a position approximately 0.5 mm below the surrounding cartilage, the optimal position of the Episealer [27]. The Epiguide is then removed and the implant is tapped into place providing a press-fit fixation onto bone.

The postoperative protocol includes protected touch weight bearing for two weeks followed by gradual progression to full weight bearing over the subsequent two weeks. Full unrestricted motion was allowed from the outset. Cycling and strength work could commence at 6 weeks building up proprioception and core control over a 6-month period before allowing return to activities tailored to the individual patient’s requirement. Patients were advised not to return to impact type sports.

### Statistical analysis

Data were collected anonymised and stored using Microsoft Excel (Microsoft, Redmond, Washington USA). All statistical analyses were performed using SPSS Ver25 (IBM, Armonk, New York USA). An a-priori power calculation was not carried out since the study aimed to include all Episealer cases. A *p* value < 0.05 was considered to be statistically significant. Descriptive statistics were used to evaluate the baseline demographic and clinical parameters. Each clinical outcome score at different time points was compared against preoperative values, using 1-sample, 2-tailed paired t-tests. Linear mixed-effects models were used to analyse the progression of outcome scores over the study period while taking into account the correlation between repeated measures. Categorical data were analysed against other variables using Chi^2^ test, and significance of variation in clinically important difference using 2 tailed Fisher’s exact test.

## Results

**Fig. 5:**
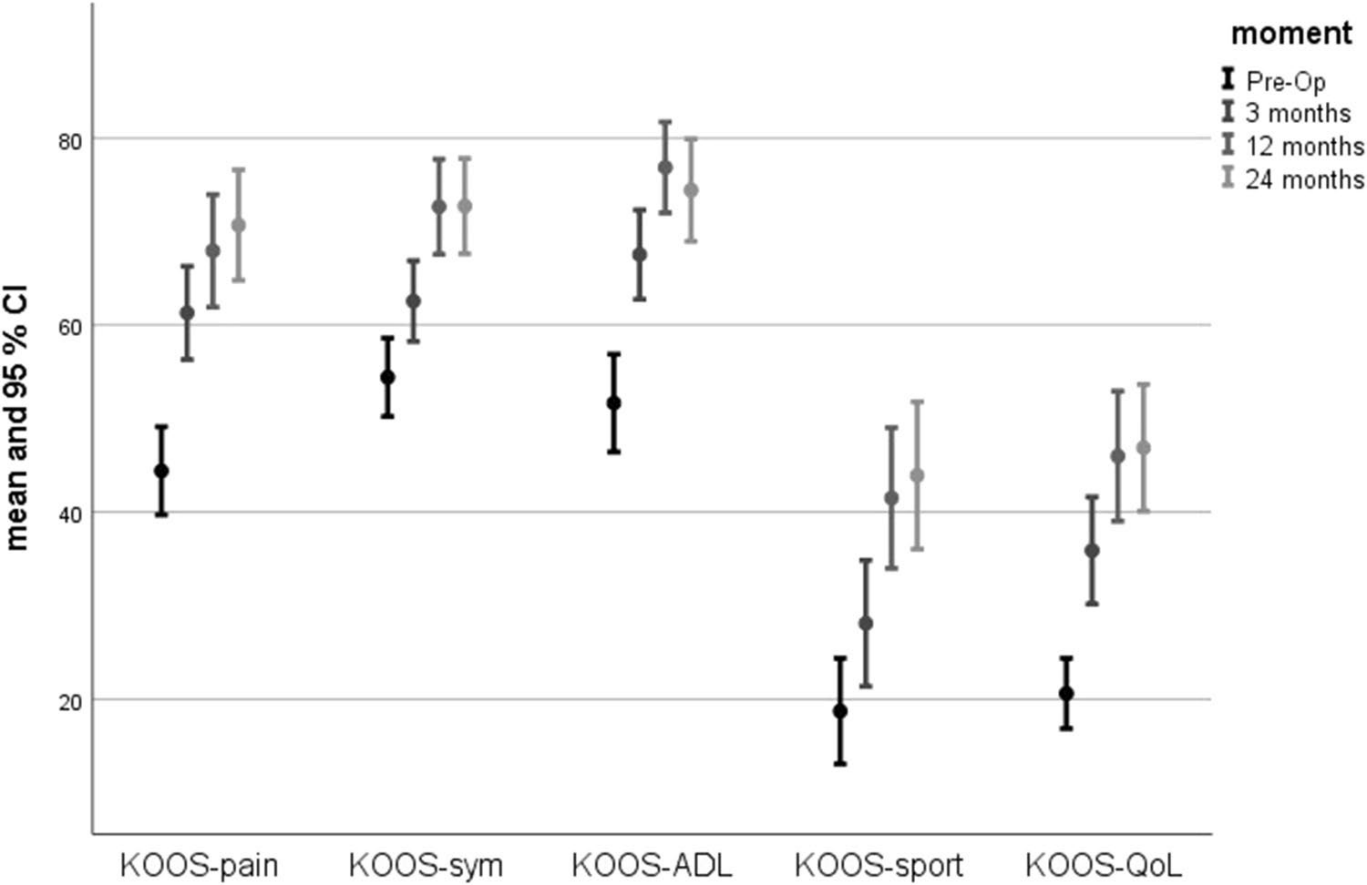
Plot with error bars chart showing KOOS domains scores values and improvement at each time point (pre-operative, 3 months, 12 months, and 24 months). All values are presented as means. The I bars indicate the 95% confidence interval. *QoL* quality of life, *ADL* activities of daily living

At 3, 12 and 24 months, knee function assessed with KOOS and VAS demonstrated sustained improvement which was statistically significant and clinically important with an increase greater than 10 points in all KOOS domains. For each domain, both linear and curvilinear trends were statistically significant (*p* < 0.0001–0.002). Significant changes seen at 3 months improved further over the study period of 24 months. In addition to the raw score results, the proportion of patients where the MCID in all domains was greater than 10 points improved from 58.3 to 72.6% at 12 and 24 months. Figure 5 shows the improvement over time for mean KOOS domain scores and 95% confidence interval.

**Fig. 6:**
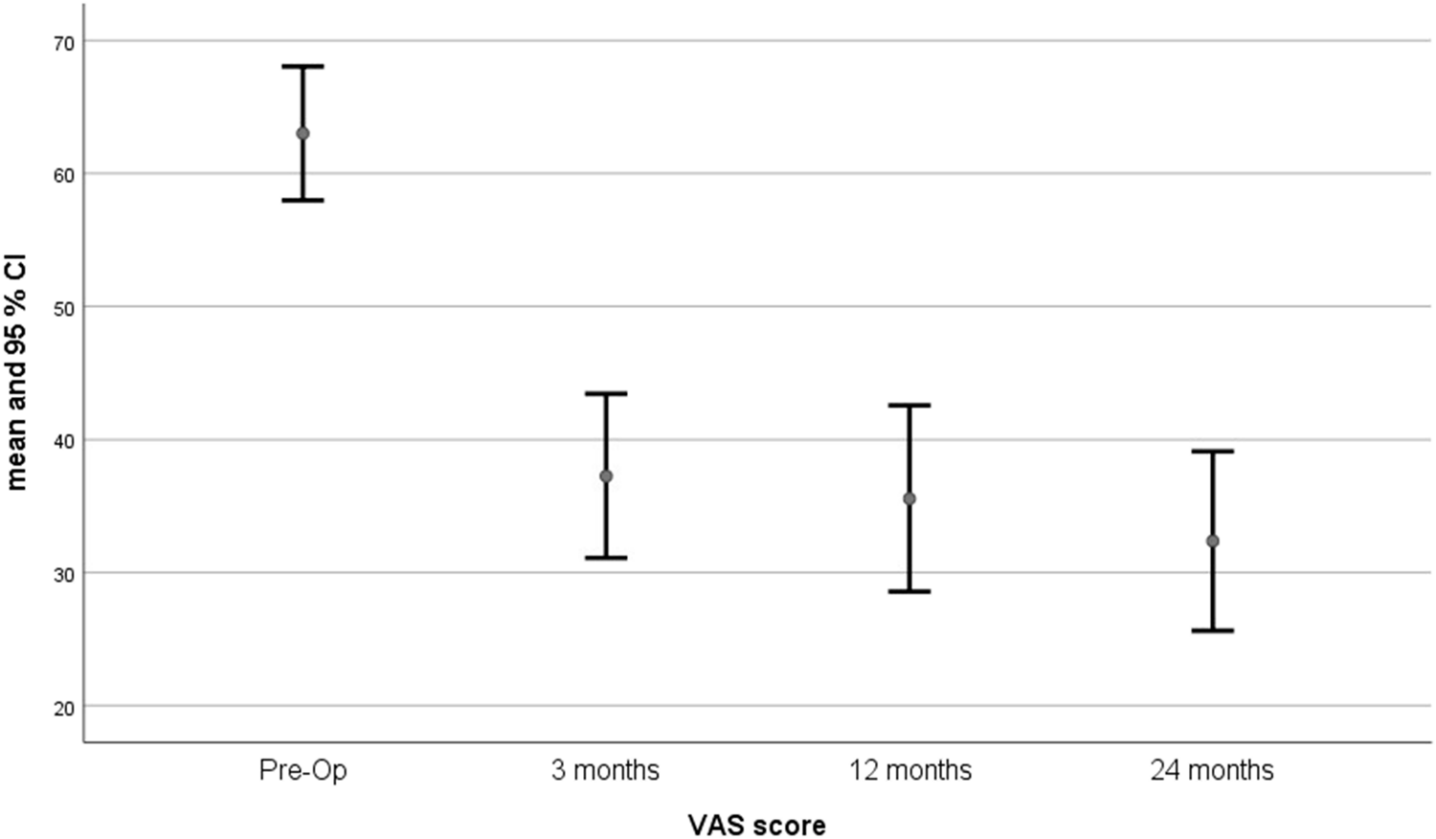
Plot with error bars chart showing Visual Analogue Scale (VAS) pain score values and improvement at each time point (pre-operative, 3 months, 12 months, and 24 months). All values are presented as means. The I bars indicate the 95% confidence interval

There was also a significant improvement in the VAS score, with a mean improvement from 63 to 36 and 32 at 1 year and 2 years, respectively (*p* < 0.001). Results are outlined in Fig. 6 with mean VAS domain score and 95% confidence intervals. Overall, the effectiveness of the intervention was consistent with a continued improvement demonstrated by a sustained clinically important difference over time (Table 3).

**Table 3:** Clinically important difference improvement from pre-operative to 3, 12 and 24 months points

Clinical outcome	Time point (months)	Mean difference	SD	95% CI	Significance level at each time point^a^	Significance level over time^b^
					*p* value	*p* value
KOOS-Pain	3	16.73	2.77	11.20–22.25	< 0.0001	
	12	22.92	3.46	16.02–29.82	< 0.0001	
	24	26.28	3.22	19.86–32.70	< 0.0001	0.002
KOOS-Symptoms	3	8.05	2.79	2.48–13.62	0.005	
	12	17.58	2.95	11.70–23.46	< 0.0001	
	24	18.3	2.81	12.70–23.90	< 0.0001	< 0.0001
KOOS-ADL	3	16.42	2.85	10.73–22.10	< 0.0001	
	12	24.75	3.28	18.20–31.29	< 0.0001	
	24	22.75	3.18	16.42–29.09	< 0.0001	< 0.0001
KOOS-Sport	3	9.47	4.1	1.28–17.66	0.024	
	12	23.06	4.53	14.02–32.09	< 0.0001	
	24	25.27	4.13	17.04–33.50	< 0.0001	0.002
KOOS-QoL	3	14.6	3.17	8.27–20.92	< 0.0001	
	12	24.05	3.76	16.54–31.55	< 0.0001	
	24	25.26	3.57	18.14–32.37	< 0.0001	< 0 .0001
VAS	3	26.31	3.22	19.89–32.73	< 0.0001	
	12	27.12	4.29	18.57–35.67	< 0.0001	
	24	30.22	3.95	22.34–38.11	< 0.0001	< 0.0001

^a^One sample paired *t* test

^b^Linear mixed effects model

**Fig. 7:**
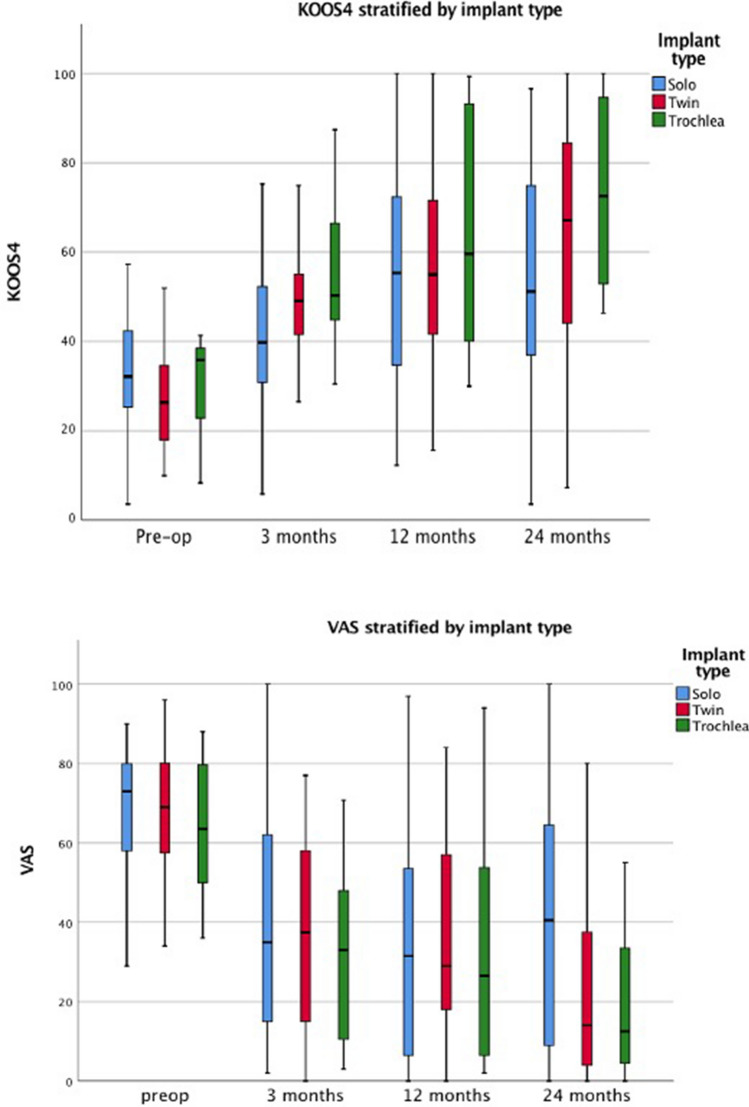
Clustered box plots showing KOOS4 and VAS pain scores categorised by implant types: condyle solo, condyle twin and trochlea at each time point (pre-operative, 3 months, 12 months, and 24 months). Values for data range on the box and whiskers are median, central distribution interquartile range and lower to upper limits. KOOS4 is calculated as the average score of the four subscale scores for Pain, Symptoms, Sport/Recreation and quality of life domains

**Fig. 8:**
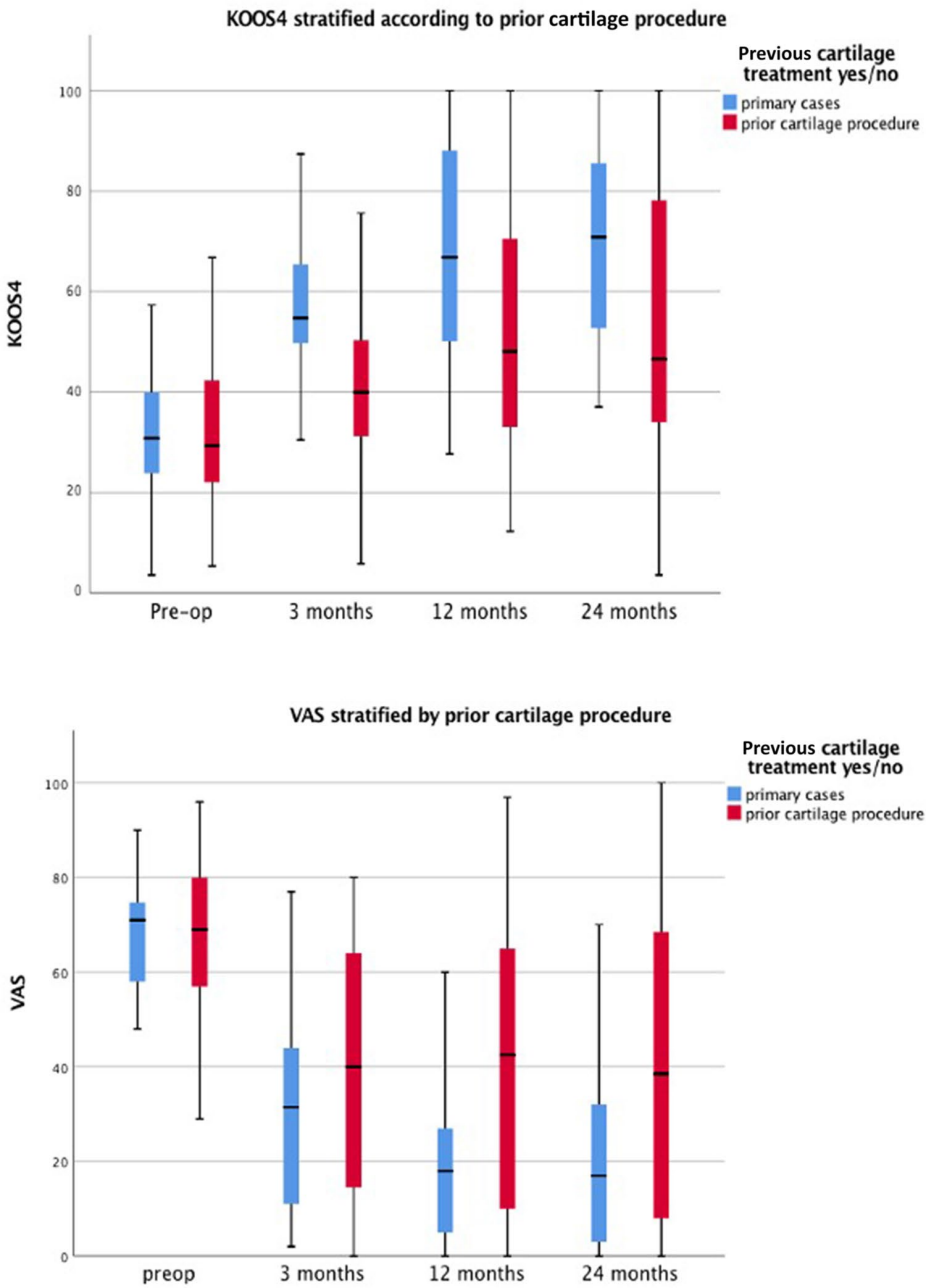
Clustered box plots showing KOOS4 and VAS pain scores categorised by primary and prior cartilage repair surgery cases at each time point (pre-operative, 3 months, 12 months, and 24 months). Values for data range on the box and whiskers are median, central distribution interquartile range and lower to upper limits. KOOS4 is calculated as the average score of the four subscale scores for Pain, Symptoms, Sport/Recreation and quality of life domains

There were no significant differences in KOOS and VAS scores between groups according to implant type (solo, twin, trochlea), or lesion size (< 3 cm^2^, 3–4 cm^2^, > 4 cm^2^) (*p* > 0.05). 48 patients (64%) had undergone prior cartilage repair procedures. There were no statistically significant baseline differences when compared to patients with no previous repair surgery, and clinical outcome results in patients who had undergone previous repair surgery were not statistically inferior when compared with patients with no history of prior cartilage repair procedures (Figs. 7 and 8). Two patients underwent arthroscopy for painful mechanical clicking and for debridement of scar tissue, with both improving, and one patient developed a DVT.

### Failures and revision procedures

Two patients underwent removal of implants during the study period resulting in a revision rate of 2.5%. The first patient had an atypical lesion with significant bone marrow oedema condyle preoperatively. The patient did not improve following implantation and re-presented with increased pain at 15 months. The implant was revised to a unicompartmental arthroplasty. In the second patient focal replacement had been performed following previous OATS cartilage repair procedure where the patient had never been pain-free. Cysts persisted around the OATS plugs and a thicker than usual Episealer implant was used for reconstruction. Symptoms did not improve, and the implant was eventually revised at 19 months to bone grafting and coverage with a chondrogide™ membrane. At the latest review, 8 months following revision the patient-reported substantial improvement in pain and function.

Beyond the study period of 24 months, one patient continued to report severe pain at their subsequent follow-up evaluation. Consequently, the focal implant was removed at 27 months and was found to be loose. Preoperative cultures in this patient suggested infection and a single-stage revision to unicompartmental knee replacement was performed, combined with postoperative antibiotic therapy. There were no further complications with this case.

## Discussion

This study is an exploratory analysis of the outcome at 2 years following partial resurfacing in the knee with an individualised mini-metal implant. The most important finding was a clinically and statistically significant improvement in KOOS score at 24 months confirming the primary hypothesis that treatment would result in improvement in symptoms and function. The VAS pain score and all subdomains of the KOOS scores were also significantly improved at 12 and 24 months. Improvement was particularly marked for the Sports and the Quality of Life domains with 25 and 26 points difference, respectively (*p* < 0.002 and < 0.001, respectively),—recognised as the most discerning KOOS domains for the assessment of treatment impact [26, 41]. No difference in clinical outcome was noted between implantation on the femoral condyle and the trochlea, and whether treatment followed previous cartilage repair surgery.

These results compare favourably with previous studies of focal articular prosthetic resurfacing. The first publication of Episealer clinical results in 10 cases demonstrated clinically important improvement at 2 years with no signs of radiological erosion of the opposing tibial chondral surface [44]. Studies following treatment with the HemiCAP and UniCAP implants (Arthrosurface. USA) have also shown improvement in outcome. Becher et al. reported the results of 21 cases with a mean 31.5 and 20.5 point increase in sports and quality of life domains of KOOS score, respectively [1]. Bollars et al. reported on 18 out of 27 patients treated with the HemiCAP implant at a median follow-up of 34 months (range 20–57 months) with good to excellent results in WOMAC, KOOS and HSS scores [2]. Dhollander et al. reported gradual clinical improvement in 14 but reported concerns regarding the longevity of clinical benefit and radiological outcomes at 48 months [8] and Laursen et al. reported a 23% 7-year revision rate following HemiCAP implantation [23].

Interim results following implantation of a hydrophilic HA impregnated metal-backed polyethylene surface implant (Biopoly implant, Schwartz Biomedical, USA) have demonstrated a statistically significant and clinically important improvement in all KOOS subscale domains [32]. However, follow-up was poor with results reported on only 12 out of 29 patients. There have been no further detailed reports on this implant as far as we are aware.

The rate of revision surgery was low (2 implants—2.5%). One was due to the progression of osteoarthritis, and in the second patient pain never improved after implantation, resulting in a revision to bone graft and collagen membrane as the tibial surface was intact. Following hemiCAP and uniCAP implantation, Becher et al. reported one revision at 2 years, one osteotomy at 2 years and one debridement at 5 years—a 13% revision rate [1]. Bollards et al. reported one reoperation with osteotomy hardware removal in 19 cases while Pascual-Garrido et al. reported a 6% revision rate at 2 years [2, 37]. Others studies suggested variable revision rates with 25–40% at 5 years [12, 23]. The Danish Knee Registry reported a 45% HemiCAP implant survival at 6 years among 230 cases and the Australian Joint registry reported 38.7% revision rate in 211 at 9 years [6, 12]. Laursen and colleagues recently reported that revisions in their long-term single-centre series occurred early within the initial 4 years period, with implants lasting beyond 5 years demonstrating a much greater longevity [24].

Alternative treatments include biological cartilage procedures and arthroplasty. Pascual-Garrido et al. compared focal CAP metallic resurfacing with biological procedures in an RCT reporting 75% success (significant improvement in all outcome measures) in CAP group and 53% in the BIO group [37]. Biological interventions required a longer rehabilitation period and provided better outcome among younger patients, whereas focal resurfacing implants allowed full weight-bearing status earlier. Rapid resumption of physical activities following biological procedures can be detrimental to the outcome [33]. It is relevant for decision making that cartilage biological procedures show significantly better outcomes in younger when compared to middle-age patients [19, 20, 42], but reduced success if prior microfracture [35]. Results of unicompartmental knee arthroplasty for uni-focal chondral lesions are unsatisfactory, suggesting that this should be reserved for bifocal bone on bone disease [14, 22, 36]. Similarly, total knee arthroplasty (TKA) is not appropriate due to the high failure rate and the inability to accommodate a return to strenuous physical activities [18, 25, 39]. TKA implant survival is reduced in active patients younger than 50 years, with a reported 9% revision rate at 2 years and 16% revision rate at 10 years [28, 34].

Mini-metal implants appear to support the surrounding articular cartilage reducing the progression of defects [13, 38]. Expansion appears to be counteracted by a well-fitting and conforming hard implant [16, 45]. Large animal models have shown that surrounding cartilage adheres to the HA coating of the Episealer implant which contributes to the longevity of cartilage [43]. These features would suggest a long-term chondroprotective effect in adequately selected patients. Conformity with surrounding articular cartilage with exact positioning of the implant to avoid high pressure is important and implants should not be inserted such that the surface protrudes. A countersunk position is preferable [5, 27]. Exacting instrumentation, detailed MRI evaluation of the affected and the opposing surfaces along with the individualised design of the Episealer implant address these issues.

There are limitations to the present study. It is a single-arm series without a control group and the follow-up period is short, however, it represents a relatively large series compared to current and past literature. In addition, all patients were consecutively enrolled and prospectively followed up with minimal loss of patients at 2 years. Radiological outcomes were not assessed and, therefore, it is not possible from this series to comment on wear of the opposing articular cartilage. Long term outcome analysis is important for mini-metal implants determining survival and duration of functional gain. Factors predictive for outcome have not been evaluated in this study but will be the subject of further longer-term evaluation.

The clinical relevance of this study is that patients with symptomatic articular surface defects that are considered inappropriate for biological options or arthroplasty can expect improvement with partial resurfacing utilising an individualised mini-metal implant.

## Conclusions

Articular surface reconstruction using an individualised mini-metal implant designed according to detailed MRI imaging, in combination with specific insertion guides, resulted in significant clinical and functional improvement at 2 years. Failure rate was low with only 2 failures before 2 years. There was no difference in improvement when performed following previous cartilage repair surgery, but the study was not powered for this secondary aim. This interim study indicates that there is a definitive place for this device in the management of a focal chondral or osteochondral defect affecting the distal femur.
